# A Mobile App to Aid Smoking Cessation: Preliminary Evaluation of SmokeFree28

**DOI:** 10.2196/jmir.3479

**Published:** 2015-01-16

**Authors:** Harveen Kaur Ubhi, Susan Michie, Daniel Kotz, Wai Chi Wong, Robert West

**Affiliations:** ^1^Cancer Research UK Health Behaviour Research CentreDepartment of Epidemiology and Public HealthUniversity College LondonLondonUnited Kingdom; ^2^CAPHRI School for Public Health and Primary CareDepartment of Family MedicineMaastricht University Medical CentreMaastrichtNetherlands; ^3^Centre for Outcomes Research and EffectivenessResearch Department of Clinical, Educational and Health PsychologyUniversity College LondonLondonUnited Kingdom; ^4^Institute of General PracticeMedical Faculty of the Heinrich-Heine-University DüsseldorfDüsseldorfGermany

**Keywords:** smoking cessation intervention, mobile, smartphone, apps, PRIME theory

## Abstract

**Background:**

Little is known about the effectiveness of mobile apps in aiding smoking cessation or their validity for automated collection of data on smoking cessation outcomes.

**Objective:**

We conducted a preliminary evaluation of SF28 (SF28 is the name of the app, short for SmokeFree28)—an app aimed at helping smokers to be smoke-free for 28 days.

**Methods:**

Data on sociodemographic characteristics, smoking history, number of logins, and abstinence at each login were uploaded to a server from SF28 between August 2012 and August 2013. Users were included if they were aged 16 years or over, smoked cigarettes at the time of registration, had set a quit date, and used the app at least once on or after their quit date. Their characteristics were compared with data from a representative sample of smokers trying to stop smoking in England. The percentage of users recording 28 days of abstinence was compared with a value of 15% estimated for unaided quitting. Correlations were assessed between recorded abstinence for 28 days and well-established abstinence predictors.

**Results:**

A total of 1170 users met the inclusion criteria. Compared with smokers trying to quit in England, they had higher consumption, and were younger, more likely to be female, and had a non-manual rather than manual occupation. In total, 18.9% (95% CI 16.7-21.1) were recorded as being abstinent from smoking for 28 days or longer. The mean number of logins was 8.5 (SD 9.0). The proportion recording abstinence for 28 days or longer was higher in users who were older, in a non-manual occupation, and in those using a smoking cessation medication.

**Conclusions:**

The recorded 28-day abstinence rates from the mobile app, SF28, suggest that it may help some smokers to stop smoking. Further evaluation by means of a randomized trial appears to be warranted.

## Introduction

Face-to-face and telephone-based behavioral support for smoking cessation can be effective [1,2]. However, uptake of these interventions is low even when they are offered free and are readily accessible [3]. Evidence suggests that Internet-based [4-7] and text messaging interventions [8,9] can be effective in aiding smoking cessation. Mobile devices have even greater potential as a flexible and cost-effective means of delivering smoking cessation interventions because of their ability to run apps that can be tailored to users’ needs and be available when needed [10,11]. Apps also have the capacity to upload data automatically onto a server for data analysis, thus making data gathering highly efficient. To date, there is only a small published study evaluating the effectiveness of a smoking cessation app [12]. This paper reports a preliminary evaluation of an app designed on the basis of a broad theory of motivation specifically designed to underpin behavior change interventions. It also examines the characteristics of users and evaluates the validity of automated data gathering as a way of screening apps for further development and evaluation.

Currently, there are more than 23,000 apps available on iTunes under the “health and fitness” or “medical” categories [13], and more than 200 of these purport to be for smoking cessation. Two recent content analyses of smoking cessation apps found that these apps did not typically adhere to evidence-based principles for smoking cessation or contain behavior change techniques (BCTs) that have been found effective in face-to-face support [14,15]. One small randomized controlled trial (RCT) has compared an app containing short messages to a text messaging system. That study found that the text messaging system produced slightly higher abstinence on one of the outcome measures than did the mobile phone app. However, it is possible that the app’s effect was masked due a small sample size (N=102) [12].

**Figure 1:**
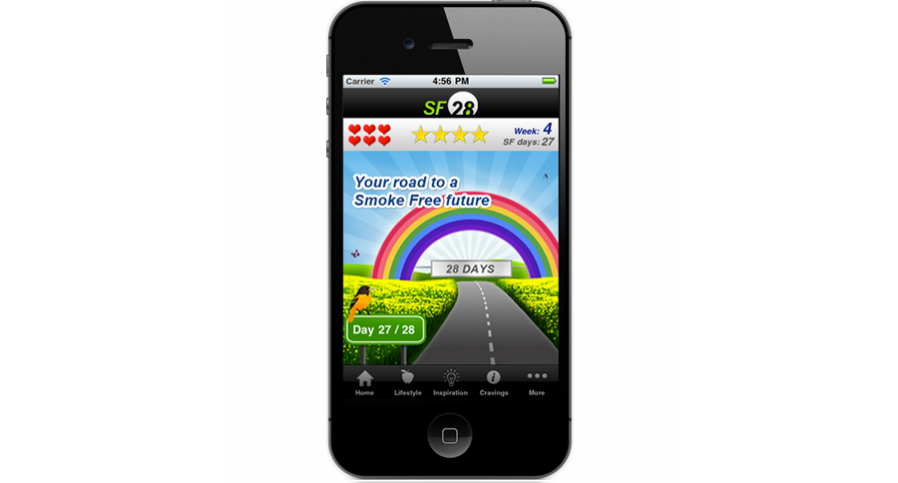
SF28 homescreen.

SF28 (SF28 is the name of the app, short for SmokeFree28) is an app that focuses on BCTs that would be expected from theory [16] and evidence [17] to aid smoking cessation (see Figure 1). The theory adopted was PRIME theory (Plans, Responses, Impulses, Motives, and Evaluations). It aims to explain and predict the impact on behavior of interventions that address higher level cognitions involving personal goals, identity, and beliefs about the harms of smoking as well as lower level drive mechanisms such as cravings, as well as the interactions between the two [18].

PRIME theory recognizes that all behavior arises “in the moment” out of the strongest of potentially competing impulses and inhibitions acting at that time. It charts potential sources of these impulses and inhibitions from learned stimulus-impulse associations, through “wants” (arising from feelings of anticipated pleasure or satisfaction) or “needs” (arising from anticipated relief from discomfort) to beliefs and self-conscious plans. It proposes that plans, such as the intention not to smoke, have to translate into sufficiently powerful wants or needs “in the moment” in order to control behavior. One factor in this is setting up clear boundaries around what is acceptable and being rewarded for staying within those boundaries. Another is doing everything possible to reduce the strengths of the wants, needs, and impulses driving the unwanted behavior.

Thus, the core of SF28 involves setting a highly salient target of becoming 28 days smoke-free and monitoring progress towards that target using the app. Maintaining abstinence for 28 days on average increases the chances of lasting success at stopping more than five-fold as cigarette cravings and nicotine withdrawal symptoms are substantially reduced in most smokers after this period [19]. The app also provides a “toolbox” of evidence-based BCTs for smokers to help them achieve the goal, including advice on the use of stop-smoking medication and licensed nicotine products, inspirational stories and videos of smokers going through the process of quitting, a distraction game, and advice on matters such as reducing exposure to smoking cues. A fuller description, including a BCT analysis of the components of the app is given in Multimedia Appendix 1 [17,20-23].

SF28 involves a registration process in which smokers provide information about themselves and their smoking history, select a quit date, and indicate whether or not they intend to use a stop-smoking medicine or licensed nicotine product. They are encouraged to open the app each day from the quit date onwards for at least 28 days. The app continues to be available after that time, but the material after that does not change. It automatically uploads users’ data including each login and whether or not they indicate that they are still abstinent. Following initial testing and gauging of user reactions, it was decided that they should be permitted up to two “lapses” and still progress towards their target during the 28 days. In this study, a “lapse” was defined as a report of having smoked any time during the intervention, “even a puff”. The app did not include any push notifications to prompt users to log in; it relied on their remembering to do so.

SF28 was developed for iPhone and made available free of charge to users via iTunes. No promotion was undertaken, so usage depended on iPhone users finding it through searches or through word of mouth. The app requested users’ consent for the use of their information in research, which is anonymous and not traceable to the participants.

Before moving to a costly randomized trial of an intervention such as a mobile app, it is important to establish prima facie evidence for its effectiveness and appeal [24]. It is also useful to be able to identify the characteristics of those who elect to use it. The fact that the app uploads data automatically offers the prospect of undertaking this exercise at minimal cost, as long as the data can be relied on. Data from prospective studies of unaided cessation and surveys of smokers who have tried to stop smoking suggest that among smokers in England (the primary target for SF28), about 15% could be expected to manage 28 days’ abstinence following their quit attempt [25-27]. This includes smokers who buy nicotine replacement therapy (NRT) over-the-counter rather than getting it from a health professional. For these smokers, the success rates are similar to unaided cessation, possibly because of inadequate usage [28]. Other evidence shows that overall abstinence rates in England in smokers who try to stop are as high as or higher than in other major English-speaking countries [29]. Therefore, one could set an a priori goal that an app should achieve a significantly higher abstinence rate than 15% for the purposes of establishing the prima facie case for further development and evaluation.

In accordance with the principles set out in the Russell Standard [30], there are grounds for specifying that the 28-day abstinence number in those using the app involve all those trying to quit, counting participants who no longer use the app as having resumed smoking. This may underestimate the quit rate if smokers who continue to abstain using the app because they do not feel they need it. However, the main alternative of including only those whose outcome is known would almost certainly overestimate success rates. The conservative approach is preferable because of the need for at least a moderate level of confidence in effectiveness before expending significant further resources on product development.

It is desirable, and in some cases essential, for evaluation of effectiveness of smoking cessation interventions to involve some form of biochemical confirmation [30]. This is because in some cases there may be significant pressure to claim abstinence falsely. In the case of the data automatically recorded from SF28, it is judged unlikely that users would log into the app so as to misreport abstinence. There would have been no human contact during the process and no social contract to pressurize a false abstinence claim. Therefore, an argument can be made that self-reported abstinence rates in an app of this kind are unlikely to be a substantial overestimate.

Given that automated outcome assessment using self-report has not been used to evaluate smoking cessation interventions before, it would be useful to assess its validity. One way to do this is to examine how closely this variable is associated with a basket of other variables that are known from previous studies to be associated with actual rates of smoking cessation. In essence, this is a measure of construct validity [31]. This approach has been used previously to identify an appropriate threshold for validating claims of abstinence using expired air carbon monoxide [32]. Variables known to be associated with abstinence are age, occupational group, and cigarette dependence [33], as well as the use of a stop-smoking medication [34].

An important variable in the evaluation of a mobile app is the extent of engagement. A simple measure of this is the number of times users open the app. There are no benchmarks for this kind of measure in smoking cessation apps, but previous research on mobile health apps has found that about one-third of app users open it no more than once and three-quarters open the app fewer than 10 times [35]. Our study could help establish a reference point for evaluation of future apps of this kind.

It is important to understand the characteristics of smokers who would be interested in using stop-smoking apps to inform further development and assess reach for different sectors of the population. One study has examined the characteristics of smokers who indicated that they would be interested in using an app for smoking cessation [36]. In that study, Internet use was not associated with social grade but was associated with being younger, more highly motivated to stop smoking, more cigarette dependent, having attempted to quit recently, having regular Internet and handheld computer access, and having recently searched for online smoking cessation information and support. This suggests that apps may have differential reach towards more dependent and younger smokers. Good data for comparison are available from a large ongoing study on characteristics of smokers in England who make quit attempts. This study, the Smoking Toolkit Study, involves monthly household surveys of nationally representative samples of people aged 16 and up [37].

From all the above considerations, the following research questions were identified: (1) Is the proportion of SF28 app users, who begin their quit attempt and record their smoking abstinence, greater than 15% (ie, is the lower bound of the 95% confidence greater than 15%)?, (2) What is the construct validity of abstinence automatically recorded for 28 days in terms of its association with variables known to be associated with success of quit attempts?, (3) What is the mean number of times the app is opened?, and (4) How do the characteristics of users of SF28 compare with smokers who made a quit attempt in the past year in a large representative sample of smokers in England?

## Methods

### Study Design

This was an observational study involving automated data collection from SF28 users between August 2012 and August 2013. Ethical approval was granted by the University College London ethics committee.

### Participants

**Figure 2:**
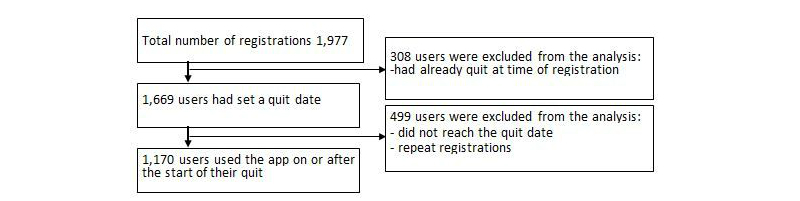
Participant flow.

Figure 2 shows the participant flow in the study. A total of 1977 registrations were recorded. Participants were included in the analysis (N=1170) if they met the following criteria: adults aged 16 years or over, smoked cigarettes at the time of registration, set a quit date, and used the app at least once on or after their preferred quit date. Those users who had already started their quit attempt at the time of registration were excluded, and if users registered more than once, only data from the first registration was used.

### Intervention

The app could be found by searching while searching for stop-smoking apps in iTunes store. Once a potential user downloaded SF28 onto their mobile device, the app would seek permission for data to be collected and used for research purposes. Each time users logged in, SF28 routinely collected data from the session. No identifying information was collected apart from the device identification (to identify multiple registrations). The terms and conditions stated “Information will be gathered concerning your use of SF28 but it shall be anonymous and not traceable to you. The information will only be used for research purposes with a view to developing improved ways of helping smokers to stop”.

When the app was downloaded for the first time, users were presented with a tutorial lasting up to 5 minutes about how to use the app. Participants were then asked to choose a quit date and to answer questions on their demographic and smoking characteristics. Each consecutive day of abstinence was “rewarded” by the app with the addition of a star on the home screen and every week with a heart. Progress towards the goal of 28 days’ abstinence was visualized on the home screen as progress down a road through the countryside towards a rainbow arch. Information on money saved to date was activated by tapping on an image of a bird sitting on a road sign saying how many days of abstinence had been achieved. More information on the app is presented in Multimedia Appendix 2.

### Measures

The primary outcome measure was the proportion of users who continued to use the app for at least 28 days (though not necessarily every day), and recorded abstinence for the full period with no more than two lapses. At the start of each session, users were asked “Have you smoked since you last used SF28” to which they responded “No, not a puff” or “Yes”. Users who opened the app at least 28 days after the declared quit date and reported abstinence up to that point with no more than two lapses having been reported prior to that were considered as meeting the criteria for success. All other users were considered to have resumed smoking. Thus the abstinence measure was a form of continuous abstinence for at least 28 days allowing for up to two reports of having lapsed.

The other variables measured were age (16-29, 30-49, or 50+), gender, occupational group (manual, non-manual, or other), number of cigarettes smoked per day as a proxy for cigarette dependence (1-9, 10-19, or 20+), time since the most recent quit attempt (never, over a year ago, or in last year), weekly expenditure on cigarettes (£1-£9, £10-£19, £20-£29, £30-£39, or £40+), and choice of medication option (none, nicotine replacement therapy, Champix—recoded as none versus any). We did not include Zyban as an option because its use in England is very rare [38]. Users then set a quit date that could be any date in the preceding 2 weeks or up to 2 weeks hence. In our analysis, users who had already started with their quit attempt at the time of registration were excluded. The number of times users browsed the app was recorded. The percentage of missing data ranged between 4 (0.3%) and 26 (2.2%).

### Analysis

A total of 1170 participants were included in the analysis. Abstinence for 28 days was calculated as a percentage with 95% confidence intervals. Construct validity of the abstinence measure was assessed by logistic regression of this measure on to the following predictors shown in previous studies to be linked to abstinence: age (positive), non-manual occupational group (positive), daily cigarette consumption (negative), and use of a stop-smoking medication (positive). Each predictor was evaluated separately because the purpose was not to model the outcome but to assess how each predictor individually was associated with it. “Usage” was defined by the mean number of times the users opened the app.

In our sample of 1170 participants, 819 (70.00%) of the SF28 users were from the United Kingdom, followed by 199 (17.01%) from the United States. We were able to compare the characteristics of SF28 users with those obtained from the smokers in England, who had tried to stop smoking in the past 12 months in the Smoking Toolkit Study (STS) [37]. The STS is an ongoing surveillance project assessing smoking and smoking cessation patterns in England. It involves a series of monthly household surveys using a sampling procedures designed to maximize representativeness. Percentages of SF28 users with particular characteristics were compared with those from the STS using chi-square tests.

## Results

The self-reported smoking cessation rate for 28 days or longer was 18.9% (95% CI 16.7-21.1). Recorded abstinence was significantly associated with older age, non-manual occupational group, and use of a stop-smoking medicine but not with daily cigarette consumption (Table 1).

**Table 1:** Associations between smoker characteristics and recorded abstinence (all odds ratios are unadjusted).

Predictor	Odds ratio (CI)	*P* value
Age category (older age)	1.66 (1.30-2.13)	*<*.001
Non-manual occupational group (vs manual)	1.45 (1.08-1.95)	.013
Cigarette consumption category	1.16 (0.95-1.43)	.152
Intended use of stop-smoking medicine	1.56 (1.16-2.12)	.003

From a total of 1170 participants, 977 (83.50%) had set a quit date on the day of registration and 193 (16.50%) had set their quit date after the day of registration. The mean number of times SF28 users opened the app was 8.5 (SD 9.0) occasions. Of all 1170 users who set a quit date, 782 users (66.84%) used the app for 2 days or more from the start of their quit date; 470 (40.17%) used it for 7 days or more; 347 (29.66%) used it for 14 days or more; 277 (23.68%) used it for 21 days or more; and 226 (19.32%) used it for 28 days or more. As would be expected, a strong positive association was found between number of times the app was opened and 28-day abstinence (OR 1.17, 95% CI 1.15-1.19, *P*<.001).

No significant associations were found between the mean number of logins between men 8.4 (SD 8.9) and women 8.5 (SD 9.0). The mean number of logins was higher for smokers aged 30-49 years (9.5, SD 9.8), non-manual occupation (9.2, SD 9.7), smokers who were taking stop-smoking medication (9.7, SD 9.8), heavy smokers (8.9, SD 9.4), and smokers who were spending £40 or more per week (8.8, SD 9.0) as compared to other users in their respective cohorts. Post-hoc analyses (using Tukey’s test) revealed that the mean number of logins was higher for smokers: (1) in the 30-49 years age group (*P*=.001) than for those in 16-29 years age group, (2) in non-manual occupations (*P*=.032) than for those in other occupational groups (retired, unemployed, and students), (3) who made a quit attempt over a year ago (*P*=.002) than for those who never made a quit attempt, and (4) who used stop-smoking medication (*P*=.001) than for those who did not use any stop-smoking medication.

Table 2 shows that compared with smokers who had tried to quit in the past year in England, SF28 users were more likely to be younger, have a non-manual occupation, be female, smoke more cigarettes per day, and spend more money on cigarettes. They were less likely to intend to use a stop-smoking medicine.

**Table 2:** Characteristics of participants compared with nationally representative sample of smokers in England who had tried to quit in the past year (all differences apart from varenicline use are statistically significant by chi-square test, *P*<.01).

Characteristics		SF28 users (N=1170)^a^ % (n)	Smoking Toolkit Study sample (N=13,706) % (n)
**Gender**			
	Female	64.49 (752)	49.44 (6776)
**Age, years**			
	16-29	50.43 (590)	34.38 (4710)
	30-49	45.38 (531)	41.23 (5649)
	50+	4.19 (49)	24.39 (3342)
**Occupation**			
	Non-manual occupation	45.64 (523)	41.86 (5738)
**Previous quit attempt(s)**			
	Tried to quit in past year	37.76 (435)	34.75 (4763)
**Medication use**			
	Nicotine replacement therapy	28.03 (328)	39.54 (5419)
	Varenicline	5.21 (61)	5.88 (806)
**Weekly expenditure on cigarettes, £**			
	1-9	8.72 (102)	20.41 (1529)
	10-19	15.56 (182)	27.74 (2078)
	20-29	19.66 (230)	21.80 (1633)
	30-39	18.97 (222)	16.50 (1236)
	40+	37.09 (434)	13.56 (1016)
**Cigarettes smoked per day**			
	1-9	19.32 (226)	32.56 (4399)
	10-19	45.90 (537)	41.26 (5574)
	20+	34.79 (407)	26.18 (3537)

^a^The percentage per variable was calculated after excluding those with missing data; thus, the “valid %” approach was used (missing data: gender n=4; occupation n=24; previous quit attempts n=18).

## Discussion

### Principal Findings

Recorded abstinence rates among users of SF28 were slightly but significantly higher than might have been expected from unaided cessation. Recorded abstinence showed expected associations with predictors of abstinence identified in previous studies with the exception of daily cigarette consumption. Compared with smokers in England who try to stop, SF28 users were younger and had higher daily cigarette consumption. They were also less likely to use a stop-smoking medication. Although the proportion with a non-manual occupation was higher than in the general population of smokers trying to stop, the difference was small.

The fact that the app was typically not used daily suggests that there is room for improvement with regard to user engagement. Users received no prompts, and it seems likely that introducing these could improve usage rates. The rate of discontinuation of app usage followed the classic relapse curve, and it seems likely that relapse was a primary driver of discontinuation. Thus, the proportion of users who logged in on or after day 28 was nearly identical to the number reporting abstinence. This would be expected given the focus of SF28, but it raises the question as to whether or not the app could be made more effective by attempting to help users recover from lapses.

It is noteworthy that the large majority of users set their quit date as the same day that they downloaded the app. Thus, it seems that apps of this kind need to be aware that many users want to get on with their quit attempt immediately, leaving minimal opportunity for planning or obtaining medication in preparation for quitting. It remains to be seen whether or not encouraging smokers to wait before quitting so that they could plan ahead would be beneficial, but it should be noted that other research has found that quit attempts made with no pre-planning are at least as likely to succeed as those that are planned in advance [39].

The overall abstinence rate among users of the app suggests that it may have helped some to achieve abstinence. The estimated effect is small, but given the extremely low unit cost of the app, it could still be cost-effective. The data provide sufficient encouragement to develop the app further and test it using a comparative trial. From the usage data, one obvious potential area for improvement would be to include push notifications to prompt users to open it every day. In addition, we did not collect data on which parts of the app were accessed. It would be useful to include this information in the next version to allow further improvements to be made. Focusing on complete abstinence for 28 days is clearly a core feature of the app, and it is not known how far this may detract from ability to help users after they have lapsed. The rationale for it was to try to set as high a bar for initial lapse as possible, but this could have come at a cost of losing users after lapse who could have been helped. This is something that merits further examination. The fact that intended medication use was lower than is found in smokers in England who try to quit may reflect the fact that many users of apps like this download them on impulse. Given the potential for such apps to support users to use medication more effectively, more attention could be given to this in future versions.

If, in the next phase of the research, it can be demonstrated in a comparative study that the app improves success rates over and above whatever else smokers might be using, this would provide a kind of “base camp” from which a program of “theory-based A-B testing” (making theory-informed changes and establishing whether this improves or worsens success rates) could be conducted as part of iterative optimization [40].

The associations between recorded abstinence and the basket of predictors mostly confirmed the validity of our outcome measure. The exception was the failure of daily cigarette consumption to predict recorded abstinence. This may have been partly because of the range restriction: the fact that the range of cigarette consumption among users of SF28 was smaller than is found in the general population, with a skew towards heavier smokers. It is also possible that the association was mitigated by the fact that medication use was more prevalent in heavier smokers (data not shown). It is conceivable that heavier smokers may be more motivated to quit, as they are more likely to experience smoking-related adverse health effects, whereas light smokers might feel less motivated to quit because of perceived lower personal risk. It would be worthwhile in future studies of this kind to identify more robust measures of nicotine dependence. Time from waking to the first cigarette of the day and strength of rated urges to smoke are two potential candidates [41].

The positive association between amount of usage and outcome is consistent with the hypothesis that the app is helpful in aiding cessation but equally could be due to smokers ceasing to use the app if they resume smoking. The association between usage and outcome should, however, be treated with caution. The problem with this association is of reverse causality, that is, smokers who resume smoking would stop using the app, and those who are performing better in terms of managing their cravings would log in more frequently.

The demographic and smoking profile of the app users may provide a useful comparator for future app evaluations. It should be noted that the app was not promoted, and so those using it had to discover it through searching on iTunes. Different user profiles would be expected with different types of iPhones and in different contexts. The tendency for users to be younger and heavier smokers was expected. It was not predicted that there would be a preponderance of women, but previous research has shown that women are more likely than men to seek support for stopping smoking and that may partly account for this result. The proportion of smokers with non-manual occupations was only slightly higher than in the general population of smokers trying to stop, which suggests that, contrary to what might have been expected, the app may have appeal across the social spectrum. The social gradient in app usage is something that merits further investigation.

### Limitations

This study had several limitations. First, there was no direct comparison condition so evaluation of whether the app helped any users to stop is based on comparison with expected unaided quit rates. However, on the conservative assumption that every user who did not log in to the app for the full 28 days had resumed smoking, the abstinence rate was slightly higher than would have been expected for unaided cessation. On the other hand, approximately a third of app users said they intended to use a stop-smoking medicine, mainly NRT, which would be expected to increase the quit rate that would have been expected had they not been using the app. Against this, it has been found that NRT, when bought over-the-counter and used without any professional support, may not improve success rates outside of clinical trials [28]. Taking all this into consideration, it seems reasonable to consider that the app may have helped some users and would have provided a useful basis for further development and for a comparative evaluation.

Another limitation is that smokers were followed up for only 28 days. It is possible that there might have been a higher rate of relapse after that point, given that the 28-day target was prominent. This will need to be addressed in a future stage of development and evaluation.

The lack of biochemical verification is another limitation. We argued in the introduction that it is implausible that users would log in to the app to record abstinence when they were smoking, and it is noteworthy that almost no one logged in and reported smoking. However, this assumption will need to be tested when it comes to a full-scale comparative trial.

The comparison of app users with other smokers trying to stop was limited to smokers in England. However, these comprised 70% of the users, and most of the remainder were from the United States where figures are broadly similar [42].

A further limitation was reliance on daily cigarette consumption as a measure of dependence. Future studies could include the heaviness of smoking index as a 2-item measure [43] or the “urges to smoke” scale [44] or both.

### Conclusions

This study provided preliminary evidence that SF28 may help some smokers to stop and that automated data collection using an app of this kind has potential to provide useful information in the early screening stage of app development. This represents a first step in an iterative process of app development and evaluation, working towards a full-scale randomized comparative evaluation of an app with a realistic expectation that the app would assist the process of smoking cessation.

## Multimedia Appendix 1

BCT analysis of the components of SF28.

## Multimedia Appendix 2

SF28: a mobile application to aid smoking cessation.

## Abbreviations

BCTs: behavior change techniques
RCT: randomized controlled trial
SF28: SmokeFree28
