# Supplementary material for: A Mobile App to Aid Smoking Cessation: Preliminary Evaluation of SmokeFree28
Source: J Med Internet Res. 2015 Jan 16;17(1):e17. doi: 10.2196/jmir.3479 (PMC4319069; doi:10.2196/jmir.3479)
Supplement: Supplementary file 1 [file jmir_v17i1e17_app1.pdf]

Supplementary materials

Appendix: 1

BCT analysis of the components of SF28

It is recommended that behavioural interventions be fully described when evaluations are reported in the literature [41].This aids the process of replication and enables those undertaking systematic reviews to compare intervention content systematically. Aside from presenting the intervention content, it is recommended that individual behaviour change techniques (BCTs) used in interventions be coded using a standard system [42]. Such a system has been developed for mobile smoking cessation applications [43] based on face to face interventions to aid smoking cessation [44], which was used to identify BCTs that were more effective [17].

The table below shows the text used in SF28 and the BCT code from Michie et al [44]

Intro Tunnel

Section	Text	BCT
Welcome	Congratulations on your decision to go for the SF28 Challenge Please give yourself 10 minutes to go through this first session	RD3
Page 1	We are confident that with the help of this App you will be able to go for a full 28 days without smoking - but it won't be easy! If it was, you wouldn't be using this App	BS3
Page 2	SF28 is your path to a smoke-free future. It is based on the latest	RC4

	scientific research.	
	Getting to be an ex-smoker is going to be the most important thing in your life for the next few weeks	BM8
	SF28 knows what challenges you will face and gives you the tools to deal with them. You set your quit day and SF28 will guide you day by day to your goal.	RC4
Why 28?	Research shows that the first 28 days after the quit date are the most important: 90% of relapses happen in this period. It is the time when the nicotine withdrawal symptoms and cravings are usually at their worst.<br><br>If you can get through this period you are not out of the woods but you have a good chance of stopping for good	BS2
Think Date	If you're going to use one of the stop smoking medicines available from your doctor, which is highly recommended, you'll need to make it at least a week away to give	BS4

	yourself time to get a prescription.	
Choose Date	If you are not getting medicine or if you already have them you can quit any day in the next 14 days. If you are ready to go you can stop today. You may want to tie it to a special day. This could be the most important day of your life!	BS4
Pick a quit date	Give yourself enough time to get ready (at least 2 days) so you can read all our advice and have time to prepare yourself. Make it easy on yourself. If you are going to a party or event in the next few days and you know you will want to smoke, then choose a date after this.	BS4<br>BS10
	Choose a stress free date. Stopping smoking can be stressful, so choose a date when you don’t have other things to worry about.	BM14
	Plan fun activities for your quit date. Keeping busy with things you enjoy will help distract	

	you from your cravings.	
Why use medicine?	There are several medicines that really can help you stop smoking.<br><br>You have probably heard of some of them. We will talk about the two main types...	A1
NRT	For convenience and flexibility you will want Nicotine Replacement Therapy. The highest dose patch is the most effective. You should top it up with gum, lozenge or inhalator. These products are safe and most people can come off them without difficulty at the end of the course.	A1
Champix	If you want a pill that can beat the cravings and are willing to see your doctor to get it, you will want Champix. This is probably the most effective medicine available. For most smokers it is very good at controlling the cravings. Some smokers feel sick when	A1

	taking it.	
Withdrawal Symptoms	Your body is used to getting nicotine. Without it, you may feel irritable, hungry, low, anxious, restless and unable to concentrate. It means that your body is recovering!	RC6
	NRT will ease these symptoms if you take enough of it. So if you are feeling bad, increase the amount you use. Most of the symptoms will only last a few weeks.	A1
Temptations	Only one thing matters: not a puff, no matter what. Every time you beat the cravings you are another step on the road to success.	BM8
	There will be lots of temptations. At first you will probably be thinking about cigarettes constantly.	BM2
	You have to hang in there. Arm yourself with things to say to yourself to stop yourself from smoking.	BS15

Smokers	Watch out for smokers. Some will wish you well but to others you are a rat leaving the sinking ship. They will try to tempt you back.	BS11
	If you can avoid socialising with other smokers for a few days then do. If not, arm yourself with what you are going to say when they offer you a cigarette: "No thanks - I'm giving up" is the easiest. But be ready for the conversation that comes next...	BS12
Stress, Parties and Arguments	Watch out for stress, parties and arguments. These are common causes of slip-ups.	BS10
	People think they can just smoke a cigarette or two - but they soon go back to smoking. Distraction is your best weapon; humming a tune in your head, breathing deeply... whatever distracts you and takes your mind off the craving.	BS15
How SF28 will guide	SF28 will guide you	RD3

you	day by day through your quit attempt. It will answer your questions, provide you tips for beating the cravings, help you get the best out of your medicine and tell you what to expect next.	
No puff	Remember - once the quit day comes there is only one thing that matters: not a puff - no matter what!	BM8
	SF28 is your guide - but remember you still have to put in the effort - you have to do the walking - you have to battle through the hard times. SF28 is your map - it is you who are making the journey.	RC4

Daily Information

Section	Text	BCT
14 days to go	Some people like to start reducing how much they smoke ahead of the quit date. That is fine, but you don't have to. The main thing is to have that date firmly in your mind as the last day you will be inhaling toxic, cancer causing smoke into your lungs.	BM6
13 days to go	Think about what it is that triggers your smoking. It may be stress at work, socialising with friends, an argument ... lots of things. Keep a note of those triggers and start to think about how you will deal with them without smoking – what do non-smokers do?	BS6
12 days to go	If you are still healthy, just for a few moments, close your eyes and imagine a doctor telling you have got lung cancer, and the effect it will have on you, your life and your loved ones.	BM13
11 days to go	Cigarettes are addictive because they	R19

	are a very rapid way of getting nicotine to your brain. Nicotine is like a parasitic worm that tunnels into your brain making you want to smoke and making you need to smoke.	
10 days to go	Nicotine acts on brain pathways that create the urge and need for you to do things. These pathways are deep within your brain. Think of nicotine as a mind-control drug - it trains your brain to need that nicotine hit	R19
9 days to go	Music can be really helpful whilst you're quitting smoking. It is a good distraction for taking your mind off cigarettes; it is also great at improving your mood. So, why not build up a collection on your iPhone for when you want to take your mind off smoking.	BS15
8 days to go	When you stop smoking you could well experience some unpleasant symptoms for a few weeks. The typical ones are: irritability, anxiety, depressed mood, hunger, difficulty	RC6

	concentrating and restlessness. These can all be controlled by nicotine products such as the nicotine patch – as long as you use them properly each day.	
7 days to go	One week to go and you may be getting a little nervous. There is no need. There is just one thing you have to remember – after D-Day – smoking is not an option – like being in a supermarket – and every day that you don’t smoke, you are one huge step towards freedom.	BM8
6 days to go	Start thinking of the money you will save. The average smoker spends £40 per week on cigarettes - £160 per month - £2000 each year!	BM10
5 days to go	Every day in Britain, 500 smokers stop and never smoke again. In 5 days time you could be one of them – you WILL be one of them.	BM8
4 days to go	In 4 days you will smoke your last cigarette. If you are using one of the stop	A1

	smoking medicines (Champix or Zyban) you should have started to take it by now. If you are using one of the nicotine products just remember – they are medicines too – and like any medicine, in order to work, you have to follow the instructions.	
3 days to go	3 days to go until your lungs and body can start recovering. Your main protection against lapsing is your steadfast determination. This will be helped by getting used to the idea of thinking of yourself as an ‘ex-smoker’; smoking is not part of your life any more.	BM8
2 days to go	With just 2 days to go, the starting line is in sight. Now is a good time to go forward one month in your mind. Just imagine how wonderful you will feel after not smoking for a month – what an achievement!	BM2
1 day to go	Tomorrow is the big day. Some people like to start from the	BS3

	moment they wake up so their last cigarette is the evening before. Many people like to stop at a given time: 6pm is a good choice. This is because evenings are often hardest and you will still be topped up with nicotine for the first day.	
Quit Date	Hooray! Today is the day your body starts recovering from the beating it has been taking from cigarettes for all these years.	BM7
	If you still have your cigarettes throw them away. They can't damage you anymore. You will feel urges to smoke for sure. But every time you resist them, you will have won another battle to be free.	BS8
Day 1	Excellent!!! You're off to a great start.	BM4
	Now you have stopped smoking, it is important to plan ahead for situations that you might find tricky. To help with this it might be useful	RD3

	to think about the coming week. New non-smokers find certain situations difficult, and these tend to fall into two categories: Social or Stressful and you can find this info in the Lifestyle section of the toolbox.	
Day 2	Well Done!! Another day without smoking. Your lungs will already be starting to recover.	BM4
	Doing something active can help with cravings. Perhaps you can escape the trigger situation for a minute or two, get a breather. Perhaps you can do something to distract yourself. Maybe you can find another way to de-stress. The nicotine inhalator, gum or lozenges are great for these types of 'just one puff' occasions.	BS15
Day 3	This is a great achievement!	BM4
	You are really progressing as a non-smoker. By now the deadly gas carbon	BM8

	monoxide will be gone from your body. Every bout of craving that you resist is another battle won.	
Day 4	Things are going really well!! You must be very proud of what you have achieved.	BM4
	Remember to make full use of the toolbox and check back for help and advice on beating cravings.	RD3
	If you are experiencing strong cravings and are using one of the nicotine products, consider increasing the dose – they are there to help you and stop you having that puff.	A1
Day 5	Five days of being a non-smoker. This can be a difficult time. The cravings can be very strong.	BM5
	The nicotine parasite will be trying every trick in the book to get you to have just one puff. Keep taking the medicine – even if you are feeling fine.	BS2<br>RD3

	To beat the urges use the toolbox.	BS14
	Slow deep breathing and light exercise can be very helpful. Just imagine how wonderful it will be when you are finally free of these cravings.	
Day 6	You are nearly at the end of week 1! That is wonderful.	BM4
	The first week is usually the worst. If you are struggling then just keep your eye on the goal – smoke-free for 28 days and then for life. Your body is well on the road to recovery – one cigarette is all it takes to undo all the good work.	BS5
Day 7 – Level 2	Well done!! You have reached level 2. Your first week as an ex-smoker!!!! Congratulations. It probably wasn't easy but your chances of stopping for good are now 10 times better than they were a week ago.	BM4

	Now on to the second week ...	
Day 8	Now is the time when the nicotine parasite will start finding new ways to get you back to smoking. It may be saying you have done really well and that you should reward yourself with a cigarette…don't!	BS2
		BM14
	Just put the idea out of your head and keep your eye on the goal – Smoke-Free for 28 days ...	
Day 9	The cravings may be less now but you might also be getting a little tired and out of sorts.	RC6
	Remember what you have achieved and when you think of cigarettes just remember that they are little tubes of cancer causing chemicals.	BM13
		BM5
	The SF-28 Toolbox contains messages from people who have been there before.	
Day 10	Day 10 and the average	BM3

	smoker will have saved £60 by now. Cravings should be getting less frequent but watch out – when they come they can catch you off guard.	
Day 11	You are well into the second week. That is a wonderful achievement.	BM4
	Don’t forget the main message: not a puff – no matter what. Keep doing what you are doing – it’s working. The days will pass and so will the weeks. In a few weeks’ time you will wonder what all the fuss was about.	BM8
Day 12	The nicotine parasite may be getting a bit desperate now. At this stage its most common trick is to tell you that you are no longer addicted and can have the occasional cigarette ... but that would take you right back to square one.	BS2
Day 13	You’re nearly half way through SF28!!	BM4

	Now you are at a stage where smokers around you are starting to smell awful, all while you begin to smell better!!	BM8
	Remember to use the cravings and inspirations tabs in SF28. There is advice in there that could help you when you feel like having a cigarette.	RD3
Day 14 – Level 3	Well done!! You have reached level 3	BM4
	Congratulations! After 14 days of not smoking you are now 15 times more likely to stop for good than when you started. It just gets easier from now on – but don’t get complacent.	BM2
Day 15	Keep this up and the curse of cigarettes will be history!	BM4
	Reminding yourself of the damage smoking does to your body will give you extra motivation to quit smoking. Cigarettes should now take up less and less of your	BM10

	thoughts but remember with just one puff you will find yourself back at the beginning having let all your hard work slip away.	
Day 16	If you are feeling that life is a bit empty without cigarettes, like you've lost a friend, look at all those millions of ex-smokers out there. Research shows that they are much happier and feel more satisfied with their lives than smokers.	RC10
Day 17	You're on the way to a smoke free future!!!	BM4
	If your confidence is a little low then just think about what you have achieved and if someone offers you a cigarette just remember that it is a tube full of cancerous chemicals – you wouldn't be allowed to give those to a dog!	BM13
Day 18	The big thing now is to build on what you have achieved. It has	BM2

	probably been hard at times – very hard. But it will get easier and before long you will be free.	
Day 19	Well done!!	BM4
	As you have reached this point without smoking, we know you will be able to build on your hard work up to now, and become a non-smoker permanently.	BM8<br>RD3
	Keep up the good work and keep using all the cravings tips we give you	
Day 20	One more day till you reach Level 3 in SF28 – your last week on the programme. The goal is in sight.	BS9
	Keep using the Toolbox and keep using your medicines. You’re getting there.	RD3
	And remember: smoking is not an option!!!	BM8
Day 21 – Level 4	Well done!! You have reached level 4	BM4

	This is a much bigger achievement than any computer game – and it is your passport to a healthier happier life.	
	Take time to reward yourself.	BS16
Day 22	What is the nicotine parasite up to now? Is it telling you that you've 'cracked it' and can have the occasional cigarette? Is it saying life is miserable and empty without cigarettes? Whatever it says, YOU are in control and you don't want to inhale cancerous chemicals any more.	BS2
Day 23	You are nearing the magic 28 days!! If you are finding time dragging a bit, there's lots to do – including free Apps on the iPhone. A really good free game is Words with Friends (and no – the SF28 team have nothing to do with it but it is fun and it is free!).	BS15
Day 24	The target is in sight – your first 28 days as an ex-smoker! Since you	BM8

	started on this journey 12,000 smokers in Britain have stopped and will never smoke again. And you WILL be one of them.	
Day 25	Nothing can stop you now – except a silly mistake. Holidays, arguments, parties, or simply boredom ... these are the danger periods for you now and one thing will keep you from smoking: the fact that you are an ex-smoker – smoking is simply not an option.	BS2<br>BM8
Day 26	Two days to go to your SF28 target!! You have done incredibly well to get here. Don't blow it now!!	BM4
Day 27	One more day till you reach your SF28 target!! You may have noticed your breathing getting easier and your skin improving. That is just the start – being an ex-smoker means a happier life and that you don't have to keep worrying when you can have your next cigarette.	BM8
Day 28 – SF28	Congratulations!!!!!	BM4

Complete	You have completed SF28<br><br>This is just the start of your new life as an ex-smoker. You will live a longer, healthier, richer, more satisfying life. Cigarettes will be a thing of the past.	BM8
Daily info post day 28	Welcome back – ex-smoker!!	BM8
	Use SF28 whenever you need to get inspirational messages from other ex-smokers and tips on how to tackle difficult situations that might arise.	RD3

If You Have Smoked

Section	Text	BCT
Smoked a cigarette	That's a shame, would you like to try again?<br>To set today as your new quit date click on try again	BM6
Try Again Now	It is great to see you still have the motivation to continue!!	BM4
	If you have only had a few puffs your chances are better than if you have gone back to smoking the same number of cigarettes as before you quit attempt.	BM3
	It is important to remember 'not a puff, no matter what' It could save your life!! Please exit the app now to start again.	BM8
Try again later	Just because you have started smoking again it doesn't mean you can't stop in the future!!	BM2
	Even though you may feel like you have failed, it doesn't mean you can't become a non-smoker in the future. You should probably leave it a while before	

	your next attempt. The App has now been reset so whenever you feel like making another attempt you will be able to start the app from the beginning. Please now exit the app.	

Lifestyle

**Section**	**Text**	**BCT**
Lifestyle	This information updates as you reach new levels, so remember to check back!	RD3
Adjusting Your Lifestyle	The information below will give you guidance on how to adjust your lifestyle as you progress through SF28	RD3
Before you quit	Coping with withdrawal Symptoms: Typical nicotine withdrawal symptoms are irritability, depressed mood, anxiety, restlessness, difficulty concentrating, hunger, difficulty sleeping and light-headedness. All of these are reduced by using one of the stop-smoking medicines available such as	RC6

	nicotine patches.<br><br>Getting through the first four weeks is the hardest. SF28 helps you though this period with the goal of being totally smoke free for 28 days.	RD3
Thinking like a non-smoker	Remember why you are quitting. Take a few seconds to imagine sitting in the doctor's office and the test results come back: The doctor tells you that you have got lung cancer.<br><br>Now take a few seconds to imagine life free from cigarettes and of not having to plan your life around cigarette breaks. Break the cycle and you will feel calmer, happier, healthier, and experience that great feeling of being an ex-smoker who has beaten it.	BM13<br><br>BM8
Changing routines	Nicotine works very cleverly to make you crave a cigarette in situations where you would normally smoke. This means that changing your routines	BS7

	is an important weapon on the battle to break free.<br><br>Now is a good time to think about when you smoke and plan to avoid as many of these situations as possible for a while.	BS3
Your social life	Some people find it helpful to tell others that they are stopping and some people don’t. It’s entirely up to you.<br><br>If you tell people, most of them will be very supportive and wish you well – even other smokers. In any event, you will have to think about what you say when people offer you cigarettes. ‘No thanks; I’m not smoking’ is a pretty good reply.	BS12
Eating and drinking	Stopping smoking will be great for your taste buds. You can look forward to food and drink tasting better as you progress through SF28. You will probably put on weight so remember to eat healthily.<br><br>Many smokers confuse	BS13

cravings with hunger so a good idea is to stock up on healthy food such as fruit so that you can snack on when you feel cravings or you are feeling hungry before meal time.

Lifestyle: Week 1

Section	Text	BCT
Coping	Some nicotine withdrawal symptoms, such as light-headedness, only last a few days. Others, such as increased appetite and urges to smoke, go on for longer. But all of them go away eventually, leaving you free of your addiction.<br><br>A lot of smokers develop a cough when they stop smoking. This is perfectly normal. It is a sign that your body is recovering.	RC6
Thinking like a non-smoker	Remember why you are quitting. Take a few seconds to imagine sitting in the doctor's office and the test results come back: The doctor tells you that you have got lung cancer.<br><br>Now take a few seconds to imagine life free from cigarettes: the smell, the money, of not planning every journey and event around no smoking signs, of not	BM13<br><br>BM8

	having to plan your life around cigarette breaks.	
Routine	If you are finding that you are craving a cigarette in particular situations think carefully about how you can avoid these.	BS7
	If you can't avoid them then plan to have something specific that you do instead. If you are using a nicotine product such as the inhaler – you can use that.	BS15
	In the first week evenings can be particularly difficult and a lot of people decide to go to bed early – that is a really good idea.	BS7
Social life	Seeing other people smoke is one of the biggest causes of craving. You may want to hang around with non-smoking friends for a while – if you can. If not, then stay inside when they go out for a cigarette. You may want to take something to do!	BS12
		BS13

	Most people who stop smoking have an increased appetite. Nicotine suppresses hunger, so when you stop smoking you may feel more hungry.	
Eat/drink	There are many things you can do, for instance eat more healthily, drink more water and do some exercise (such as walking).	BS13
	Remember that alcohol lowers your inhibitions, so drinking can be risky for you in the first couple of weeks after stopping.	BS1

Lifestyle: Week 2

Section	Text	BCT
Coping	Many people find they have difficulty concentrating for the first couple of weeks of not smoking. If you are using one of the nicotine products you can avoid this by making sure you use enough. We recommend a patch plus one of the faster acting products such as inhaler or gum. If you are just using one product and are feeling bad, try adding another – it does work and is perfectly safe.	RC6
Thinking like a non-smoker	For most smokers the first week is the hardest but sometimes the first week is easy and you suddenly get hit in the second week. The novelty of quitting has worn off and it all seems like a big effort. You need to keep up your morale. Just look on this as part of your journey to freedom. Tomorrow is another day and as long as you don’t smoke it will be a better one.	BM8

Routine	Now is a good time to look at your routines again and see whether they are helping you or making it harder to stay off cigarettes. If you can't avoid situations when you would have smoked then make sure you have something else you can do instead.	BS7
Social life	If smokers are still offering you cigarettes, now that you have gone a week without smoking you may feel more comfortable saying: 'No thanks – I've gone a week without smoking and I don't want to go back to it now'.	BS12
Eat/drink	You may want to snack on glucose tablets. They are very cheap and easily available from newsagents and chemists and they do not make you put on weight - surprisingly. A whole packet of glucose tablets has no more calories than a slice of bread!	BS13

Lifestyle: Week 3

Section	Text	BCT
Coping	Very few smokers desire a cigarette when they know they can't smoke, e.g. on a plane journey. One way to beat the cravings is to remind yourself that smoking is not an option for you – it's not going to happen.	BM8
Thinking like a non-smoker	Stopping smoking is a battle against the nicotine parasite. You are winning the battle and every day that goes past it will get weaker and you will get stronger – but only as long as you do not have a single puff on a cigarette. Keep using your medicines and remember that you are an ex-smoker and smoking is not an option – no matter how you feel or what happens.	BM8
Routine	As things start to get easier, you may feel able to move back to some of your old routines. That is fine, but be careful! And be ready to switch again if you need to.	BS7

Social life	Now that you are in your third week of not smoking, your smoking friends may be feeling a little bit envious. And your non-smoking friends will be delighted for you.	BS12
	Your main problem now is just that moment of weakness - it is so easy to just take a cigarette and go right back to square one. But remember: you don't smoke any more.	BM8
Eat/drink	You will probably be noticing some weight gain now. That is perfectly normal. Just remember to eat healthily and do as much physical activity as you can. For example, walk up stairs rather than taking lifts. Try to make sure you walk for at least 20 minutes a day.	BS13

Lifestyle: Week 4

Section	Text	BCT
Coping	If you find yourself craving a cigarette, imagine how you will feel, of you smoke one and undo all the work you have put in so far. Imagine the cancerous chemicals in the smoke destroying the DNA in your lungs. Could that cigarette be the one that finally triggers cancer? But if you resist the craving, you will have won another battle against the nicotine parasite. And every battle you win weakens its power.	BM13
Thinking like a non-smoker	You are so close to success and your new life as an ex-smoker. Many ex-smokers look back with fondness on smoking – that is fine. But that is in the past – you have moved on to a new and better phase in your life. Not smelling of tobacco, not wasting money on tubes of cancerous chemicals, not having to worry about blowing smoke in the face of people you are talking	BM8

	to ... freedom.	
Routine	You are nearly at the end of your SF28 journey. Things are likely to be a lot easier than when you started but you are not out of the woods. Things that triggers smoking can come out of the blue so be ready for them.	BS2
	Remember that there are loads of tips of how to beat cravings in the SF28 Cravings toolbox.	RD3
Social life	If you have been keeping quiet about trying to stop smoking, you can now feel more confident about telling people that you have stopped. Your chances of staying off for good are 20 times higher than they were when you started.	BS12
Eat/drink	One way of telling whether you are genuinely hungry or just fancy some tasty food is the ‘apple test’. If you wouldn’t eat an apple then you aren’t really hungry. And if you are not truly hungry and you are concerned about your weight, don’t eat.	BS13

Lifestyle: Ongoing weeks

Section	Text	BCT
Coping	Most nicotine withdrawal symptoms will have gone by now, but some, such as increased appetite, can last longer than 28 days. These do eventually go away though and you will feel so much better and happier than you did when you were smoking.	RC6<br>BM8
	The main relapse triggers to watch out for are: stress, holidays, parties and boredom! Your main defence is that you are an ex-smoker now – smoking is not an option.	
Thinking like a non-smoker	You've won the SF28 challenge and now there is no reason to go back to smoking.	BS2
	The nicotine parasite will still be lurking though – ready to strike in moments of weakness – so watch out!	BM8
Routine	You are now well established in your life	BS11

	as an ex-smoker. As far as routines go the main things to watch out for are celebrations, parties and anniversaries. You must go to these prepared to resist offers of cigarettes.	BM8
	You are an ex-smoker and smoking is not something you do any more.	
Social life	If you are using one of the faster acting NRT products (like gum, nasal spray, lozenges or the inhalator) then make sure you have these to hand and be prepared to use as much as you need to get through social situations that trigger cravings. If not, try and get hold of some - they are helpful and safe to use with other stop smoking medicines.	A1 BM8
	Remember the 'Not a puff, no matter what' rule. This is the most important rule in your life at the moment. Sometimes it will seem easy to break it but doing that could cost	

	you your life.	
Eat/drink	Now that you have finished the SF28 challenge you can start to think about making sure you keep to a reasonably healthy diet. If you are hungry between meals then eat fruit (or perhaps the occasional chocolate and bun!); make sure you always have vegetables with your meals; keep your alcohol levels to an average of 3 drinks per day for a man and 2 per day for a woman. And do remember to stay active .	BS13

Inspirational Stories

Section	Text	BCT
1	Hangovers aren't nearly as bad once you stop smoking!' Matthew, London	BM5
2	Having stopped, I would never go back - my hair and clothes don't smell of cigarettes anymore, I don't get breathless and cough, and the money saved is phenomenal. Best of all, it means I know that I've done one of the most significant things I can do to improve my health, which I hope means I'll live longer and be healthier.' John, Birmingham	BM5
3	Food tastes better, I feel fitter and my clothes don't smell of stale smoke.' Ahmed, Cardiff	BM5
4	I smoked for 30 years, then at the age of 50 had 2 heart attacks, I stopped smoking immediately, and feel better all round. The cravings don't go away but it gets much easier.' Julie,	BM5

	Manchester	
5	It's not easy but persevere with it, the health benefits and how you feel in the long term are well worth it.' Molly, Kent	BM5
6	Life has been a lot healthier and will probably be longer. Don't forget - the cravings will pass.' James, Newcastle	BM5
7	Quitting smoking was the best thing I ever done, now I get to spend more time with my family.' Nicole, London	BM5
8	I packed up smoking completely in 2004 and have saved myself about £3000 up to the end of 2010.' Frank, London	BM5
9	I was diagnosed as suffering from C.O.P.D. and had found it hard to breathe and was told if I did not give up smoking I would die within a year so I wanted to give up and did. This was over 10 years ago and I 'm still waiting to die!' Peter, London	BM5
10	I stopped smoking over two years ago and I	BM5

	gave up because I was losing my voice and I had to have a biopsy on my throat and they found out there was something there. It scared me so I went to the chemist and got the 24hour patches and never looked back! I am glad to say I got me voice back and I feel a lot better for giving up Smoking. I'm really pleased with myself too.' Steve, Glasgow	
11	Stopping smoking has changed my life.' Beth, Norfolk	BM5
12	3 days of hell, 3 months of craving but then nothing really, just the odd pang.' Lucy, Cambridge	BM5
13	I stopped because of health problems, and stopping smoking has improved my health to some degree as well as improving my taste and smell.' Kevin, Nottingham	BM5
14	Food tastes great, I'm now healthier and able to play with my children.' Shawn, Leeds	BM5
15	I feel much healthier.' Richard, S. London	BM5

16	Since I gave up I have been healthier had more money to spend on holidays etc. Plus, I don't smell like an old ash tray!' Valerie, Edinburgh	BM5
17	It's easier than you think, and the money I saved paid for a holiday.' Victor, Coventry	BM5
18	Easier to do than I thought, everything smells so much better now.' Stacey, Bristol	BM5
19	I feel fresher and more in control - not constantly craving nicotine.' Matthew, London	BM5
20	I no longer have to think about where can I go to have my next smoke or when it will be, especially having to go outside at restaurants or at work to smoke. I feel healthier, get out of breath only when running for a bus and feel less anti-social.' Raj, Bradford	BM5
21	It was hard to begin with and something I had wanted to do for a long time but, after a couple of months,	BM5

	found I had a lot more money, both in my purse and in the bank. I feel healthier, food tastes better and I am fitter and, since quitting over four years ago, have been able to afford take a proper holiday every year.' Geoff, Southampton	
22	I found that when trying to get fit it was too hard so it really helped quitting. Susan, Warrington	BM1
23	I gave up when I found out I was pregnant with my first child, and never smoked again. It’s been over 10 years now, and I most certainly don't regret it. If you can't do it for your kids, who can you do it for? Jacci, Surrey	BM10

Craving Tips

Section	Text	BCT
1	Think back to just one achievement in your life - then to another - then to another. Now think how you will feel when you have ditched smoking.	BM8
2	A quick burst of exercise can get rid of cravings. Exercise helps because it: Reduces the stress caused by wanting a cigarette. Makes you feel happier which reduces cravings. Directly reduces cravings by providing a different chemical to your brain. It need only be a few knee bends, a short walk or even just tensing and relaxing the muscles in your legs.	BS15
3	It is best to plan activities in advance to fill the times when you would normally have a cigarette. Spend a few seconds thinking of things you could do.<br><br>If you can't think of something right now, you could talk about it with a friend or member of your family. And you could even ask them to do the	BS15<br><br>A2

	activity with you.	
4	Make sure your mental energy stays high so you can deal with the cravings: Give yourself time to relax doing something you enjoy if you can. This will help maintain the energy you need.	BS10
5	Quitting smoking requires a lot of effort - a lot of mental energy. Mental energy is like physical energy: Both run low when you use them a lot. After running low, you need to rest to recover that energy.	BS10
6	Here is another technique which you can try: When you have an urge to smoke, imagine yourself sitting in the doctor's office getting the devastating news that you have got lung cancer - and now imagine telling your family	BM13
7	Just think - if you give in and have a cigarette it could be the one that triggers lung cancer - or it could be the one after that - or the one after that - and one cigarette will lead to another.	BM1
8	Another way to deal with	BM2

	cravings is to boost your motivation. It may seem silly but say to yourself 'Today I will not smoke - no matter what'	
9	Relaxation can really help with cravings. Concentrate on your breathing and breathe in and out, deeply and slowly - and relax your muscles with each breath	BS14
10	When craving strikes, always do something - don't just wait for it to go away. Can you get out of the situation? Can you pick up something to fiddle with? Can you get a glass of water? Every bout that you resist is another victory.	BS15
11	Try really tensing the muscles of you legs for 5 seconds, then relaxing them, then tensing them again and relaxing them. Do this for a minute or so. Research has shown that this can reduce cravings.	BS14
12	Remember that people can smell smokers as soon as they enter a room. And it's not pleasant. You are free from that now. One puff and you'll be back where	BM13

	you started.	
13	When the craving hits and you really want a cigarette, imagine your body returning to how it was when you were a smoker. Your lungs blackening and all the health risks returning. Then think of how it is now – having a great chance to heal itself. And improving all the time.	BM8
14	Quitting smoking is like a battle. You are winning. When you have a strong craving: Visualise the battle between you and the cigarettes. You are not going to let them win. Stay strong. Create the image in your mind of crushing that cigarette.	BS15
15	Chewing glucose tablets can help beat the cravings. Keep a packet handy. You can chew lots of them because they don't contain many calories.	BS15
16	A lot of smokers find that going to bed early helps escape the cravings early on - they are often worse in the evening.	BS11
17	Take time to reward yourself. Do something	BS16

	you enjoy which doesn’t make you think of smoking: Spend time with your family or friends. Watch your favourite TV programme or a film. Go out to your favourite place.	
18	Remember that the craving is just the nicotine parasite trying to get you back to smoking - sucking on a cigarette is inhaling this parasite so it can worm its way into your brain.	BM13
19	If you know some tunes you like, sing them to yourself in your head over and over until the craving goes away.	BS15
20	When the going gets tough, focus on the prize: smoke-free for 28 days. You can do it. You must do it.	BM2
21	There is only one thing that matters. Not having that puff on a cigarette. For you it is not an option - imagine you are in a supermarket - you don't want to smoke because you can't.	BM8
22	Every time you resist the cravings, you have won another battle. It won't be long before you have won the war.	BM2

23	Cravings can hit you out of the blue. Watch out for them and just remind yourself - smoking is not even an option.	BM8

Medicine

Section	Text	BCT
Gum	Gum comes in two strengths high (4mg) and low (2mg). It's generally best to go for the stronger gum. Chew it regularly, not just when you crave.<br><br>Pros: Flexible to use. They can be used more frequently to combat very strong cravings.<br><br>Cons: They have a strong taste, which takes a while to get used to. Requires regular chewing (10-15 pieces a day), which may not always be easy. Best to use alongside the patch.	A1
Nicotine Patch	These are available as either a 16 hour patch, or a 24 hour patch. Patches are applied to the skin in the morning and removed at the end of the day (16h) or the next morning (24h).<br><br>Pros: Very simple to use<br><br>Cons: They deliver nicotine slowly, so can't be used to help with particularly strong	A1

	cravings. If used overnight they can affect your dreams. Best to use them with one of the faster acting products.	
Inhalator	An inhalator is a small plastic tube containing a nicotine cartridge. The nicotine from this product is absorbed through the mouth rather than inhaled into the lungs (despite the name).<br><br>Pros: Provides the same action as smoking a cigarette (hand to mouth).<br><br>Cons: The inhalator needs to be puffed on for 20 minutes each hour and can irritate the throat. Best to use with the patch.	A1
Nasal Spray	This uses a fine spray to give you nicotine through your nose. It is quickly absorbed and starts to work within ten minutes.<br><br>Pros: Fast acting, removes withdrawal symptoms quickly.<br><br>Cons: Gives burning sensation in the nose	A1

	when you first use it but people get used to it. Works well when used with the patch.	
Champix	Champix contains varenicline tartrate. This works on the part of the brain that causes nicotine craving. It’s activated whenever you have a pleasurable experience (such as eating nice food).<br><br>It is very effective in reducing cravings and is probably the most effective stop-smoking medicine available. Some concerns have been expressed about mood disturbance as a side effect but these have not been found in large well-designed studies.	A1
Lozenge	These are tablets that you suck for approximately 30 minutes. They come in different strengths - 1 or 2mg if you are less dependent, and 4mg if you are more dependent.<br><br>Pros: The 4mg lozenge is fast acting and reduces cravings within minutes.	A1

Cons: For best effects lozenges should be sucked regularly across the day (12-15 tablets) which may not be easy. Good to use with the patch.

About

Section	Text	BCT
Paragraph 1	SF28 has been developed by experts to help you stop smoking. It sets up the challenge of being completely smoke free for 28 days. That is the period when craving and withdrawal symptoms are at their worst and if you can make it through without a single puff on a cigarette you are 20 times more likely to stay off cigarettes for good.	RD3
Paragraph 2	Robert West is the main designer behind SF28. He is a psychologist who has been researching how best to help people stop for 30 years during which time major advances have been made. For more information go to www.rjwest.co.uk.	A5
