# Supplementary material for: A Mobile App to Aid Smoking Cessation: Preliminary Evaluation of SmokeFree28
Source: J Med Internet Res. 2015 Jan 16;17(1):e17. doi: 10.2196/jmir.3479 (PMC4319069; doi:10.2196/jmir.3479)
Supplement: Supplementary file 2 [file jmir_v17i1e17_app2.pdf]

Appendix: 2

SF28: a mobile application to aid smoking cessation

Rationale

SF28 takes smokers through the first 28 days of a quit attempt – helping to maintain their resolve by setting a clear goal, monitoring progress to that goal, and providing advice to help avoid and cope with cravings.

Aim of SF28

To increase the probability of success of quit attempts.

Underlying theory

Smoking is driven by a variety of factors (1): a) nicotine establishes impulses to smoke in situations where smoking often occurs; b) it causes the smoker to want to smoke because of anticipated enjoyment; c) repeated exposure to nicotine from smoking causes a feeling need to smoke (a kind of 'nicotine hunger') when CNS concentrations become depleted; d) it also results in a range of other nicotine withdrawal symptoms during abstinence (e.g. irritability, depressed mood, increased appetite) (1) which are relieved by smoking; e) smokers develop a false belief that smoking helps with stress which creates a need to smoke in stressful situations; f) some smokers also develop a strong identity as a smoker which they value; g) some smokers also enjoy the social interaction with other smokers.

The main drivers of smoking become significantly reduced after a month of total abstinence: particularly the nicotine hunger, cue-driven impulses to smoke and nicotine withdrawal symptoms. This means that if someone is able to stop smoking completely for one month, his or her chances of becoming a permanent ex-smoker are increased fivefold (2).

To sustain abstinence (3), resolve to achieve this must be stronger than urges to smoke at all times when the opportunity to smoke is present. Using concepts contained within the PRIME Theory of motivation (4) (5), stopping smoking involves maintaining the desire and capacity to inhibit smoking sufficiently high to override impulses to smoke that can arise at any time following the start of a quit attempt. This involves:

1. Minimising frequency, duration and strength of impulses to smoke by:
   a. Reducing desire to smoke by: i) countering beliefs about the functional value of smoking, e.g. that it provides stress relief; ii) appropriate use of stop-smoking medications such as nicotine replacement therapy or varenicline; iii) development of strong 'ex-smoker identity' in which being a smoker is not valued
   b. Establishing behaviours and/or thought process that

distract attention from the desire to smoke

c. Reducing exposure to stimuli that trigger the desire and/or directly create the impulse to smoke

2. Maximising the desire not to smoke by:
   a. Establishing a strong 'personal rule' of 'not a puff no matter what' as part of a new ex-smoker identity
   b. Maximising the salience of negative emotions at the prospect of smoking, e.g. by concern over health, concern over losing the gains made thus far in the quit attempt, shame and/or embarrassment at having failed in the quit attempt
   c. Maximising positive feelings arising from the benefits of not smoking, e.g. feeling proud of achievement, experiencing praise from others, experiencing satisfaction at observable markers of health improvement
3. Maximising the translation of desire not to smoke into inhibition by:
   a. Gaining experience of successful self-control
   b. Reducing influence of factors that undermine capacity for self-control, e.g. stress, tiredness, drinking alcohol

Because of the pharmacological actions of nicotine as delivered by cigarettes, the strongest imperative is to prevent any lapse at all. If a lapse does occur, there is a still a chance of recovery but it is small.

Principles underpinning SF28

SF28 seeks to help smokers to achieve 4 weeks of total abstinence to set them on the road to life as an ex-smoker. It focuses on behaviour change techniques (BCTs) that are expected to play the most significant role and can be delivered by means of a mobile.

The application is built around: a) providing a clear goal for the quit attempt and maintaining the salience and reward value of making progress towards that goal; b) a strong focus on development of an ex-smoker identity to make the idea of having a cigarette unthinkable; c) the use of evidence-based methods of reducing desire and impulses to smoke, such as nicotine replacement therapy and light exercise.

The core of SF28 is the target of 28 days smoke free and monitoring progress towards that target using the mobile. To help achieve that target the smokers is provided with a 'toolkit' of activities and aids. These include: a) advice about stop-smoking medications, which one to use and how to use it effectively; b) advice on changes to lifestyle to reduce exposure to smoking triggers and maintain mental energy needed to exercise self-control; c) inspirational statements from smokers who have stopped to bolster positive motivation; d) advice on specific activities to minimise or help cope with cravings when they occur.

The 'look and feel' of the application is designed to make the experience of using it enjoyable and involving

minimum of effort. Attention has been paid: a) to the overall design and use of attractive imagery; b) to minimising habituation to the imagery while maintaining a reassuring continuity; c) to providing a clear structure and strong sense of forward direction to the goal.

From the Smoking Toolkit Study one would expect 15% of smokers who try to quit to succeed for 28 days if they use no form of evidence-based support (such as the NHS stop-smoking services). Using a conservative assumption that everyone who did not actively log in and record themselves as abstinent for at least 28 days had resumed smoking, 20% of those making a quit attempt with SF28 succeeded for that duration. While the estimated effect is small, the cost-effectiveness would be high in terms of cost per life years expected to be saved.

A controlled trial is warranted to gain a better estimate of effect size.

References

1. West R. The multiple facets of cigarette addiction and what they mean for encouraging and helping smokers to stop. *COPD.* 2009;6(4):277-283.
2. West R, Stapleton J. Clinical and public health significance of treatments to aid smoking cessation. *European Respiratory Review.* 2008;17:1-6.
3. Michie S, van Stralen MM, West R. The behaviour change wheel: A new method for characterising and designing behaviour change interventions. *Implement Sci.* 2011;6:42.
4. West R. *Theory of Addiction*. Oxford: Wiley-Blackwell; 2006.
5. Michie S, Hyder N, Walia A, West R. Development of a taxonomy of behaviour change techniques used in individual behavioural support for smoking cessation. *Addictive Behaviors.* 2011;36(4):315-318
